# Supplementary material for: Ultrabroadband high-resolution silicon RF-photonic beamformer
Source: Nat Commun. 2024 Feb 16;15:1433. doi: 10.1038/s41467-024-45743-9 (PMC10873374; doi:10.1038/s41467-024-45743-9)
Supplement: Supplementary file 1 — Supplementary Information [file 41467_2024_45743_MOESM1_ESM.pdf]

Supplementary notes for:

Ultrabroadband high-resolution silicon RF-photonic beamformer

Pablo Martinez-Carrasco[1], Tan Huy Ho[2], David Wessel[2] and José Capmany[1]

[1]*Photonics Research Labs, iTEAM Research Institute, Universitat Politècnica de València, Valencia, Spain*

[2]*Ottawa Wireless Advanced System Competency Centre, Huawei Technologies Canada Co., Ltd., Ottawa, Canada*

Supplementary Note 1.

Origin of beam-squint in the un-equalized true time delay beamformer architecture

In their work, Zhu et al. proposed an antenna spacing, *d,* of *λ/2* for a frequency of 16 GHz, i.e. 9.4 mm, and a temporal resolution of 2 ps, which is equivalent to what we have referred to as the basic unit delay **U**. For the established value of *d*, it is easy to verify that *d/c < 32 ps,* which corresponds to the delay value for the seventeenth pointing angle. This is the reason why only 16 frequency-independent angles appear.

By applying the equations developed in Methods, we can study when frequency-dependent solutions begin to appear in the Array Factor.

$$\frac{d}{c}\sin(\theta) - BU = -\frac{1}{Nf} \tag{S1}$$

In this case, the first pointing angle observed in the region of negative angles for low frequencies corresponds to the maximum delay between antennas, which is 31U.

$$f = \frac{1}{\frac{d}{c}\sin(\pi/2) + 31U} = 10.7GHz \tag{S2}$$

Supplementary Fig.1 shows the beam patterns for lower frequencies and for this frequency, showing that negative solutions begin to appear.

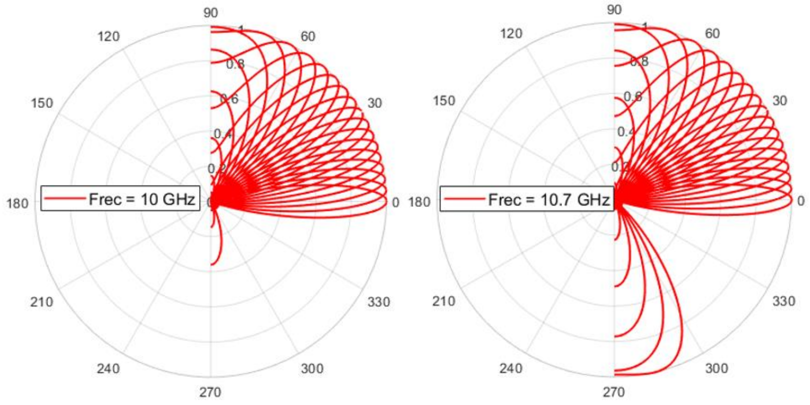

a) b)

Supplementary Figure 1. Beginning of the emergence of low-frequency negative pointing angles in the previous architecture. a Beam patterns for 10 GHz. **b** Beam patterns for 10.7 GHz. From this second frequency the lobe corresponding to the delay between 31U antennas starts to appear at -90º.

Below a frequency of 10.7 GHz the system is not capable of targeting any negative angle and it is at this frequency that these angles start to appear. In the same way, we can determine from which frequency we can recover the complete 32 pointing positions, even though half of them present beam-squint.

$$f = \frac{1}{\frac{d}{c}\sin(\pi/2) + 16U} = 15.8\,GHz \tag{S3}$$

This is demonstrated in Supplementary Fig. 2, which shows how for frequencies below 15.8 GHz the 32 pointing angles do not appear, taking into account that half of them are frequency dependent.

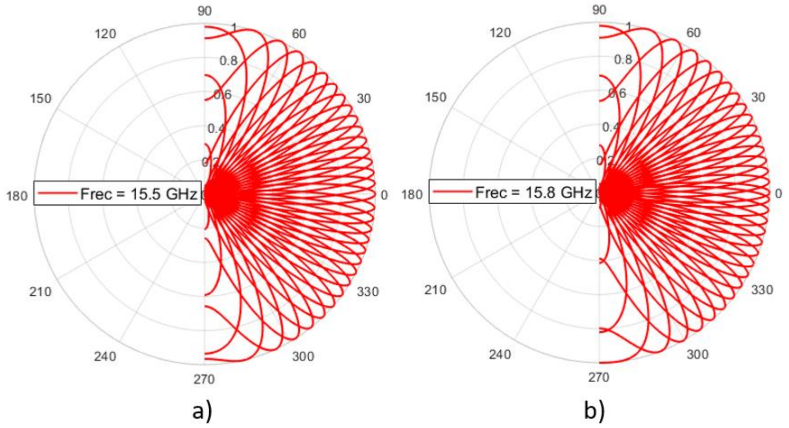

Supplementary Figure 2. Beginning of appearance of all pointing angles for high frequencies in the previous architecture. a Beam patterns for 15 GHz, only 31 pointing angles are displayed. **b** Beam patterns for 15.8 GHz. We recover all the 32 pointing angles.

At 15.8 GHz and above, 32 positive and negative pointing angles are displayed. Notice that the last pointing angle is really close to the broadband position and this distance becomes smaller with increasing frequency. It is also possible to determine the maximum frequency at which solutions with beam-squint begin to overlap with frequency-independent solutions in the region $[0, \pi/2]$, as shown in Supplementary Fig. 3.

$$f = \frac{1}{\frac{d}{c}\sin(0) + 31U} = 16.1\ GHz \tag{S4}$$

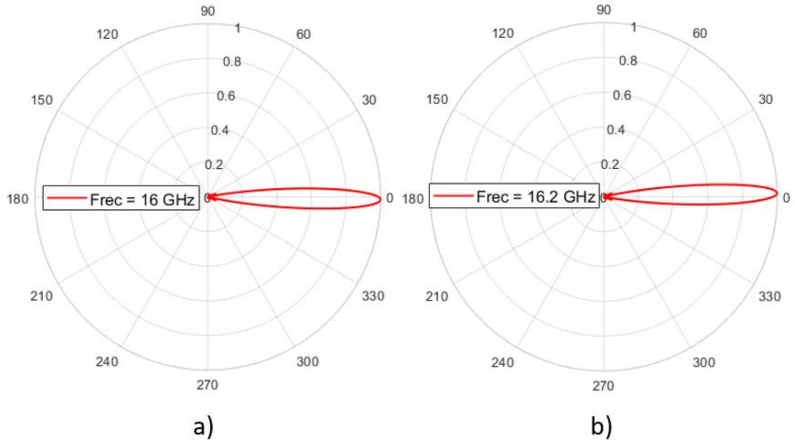

Supplementary Figure 3. Overlapping of the pointing angle with other array configurations. a Beam pattern corresponding to 16 GHz. **b** Beam pattern corresponding to 16.2 GHz. In the second figure we can appreciate how the pointing angle is beyond 0º, thus overlapping with other array configurations.

This results in a limited set of frequencies, ranging from 15.8 GHz to 16.1 GHz, in which we find 32 different pointing angles that deceive us, promising to be frequency-independent but exhibiting beam-squint behavior.

Supplementary Note 2.

Equalizer stage design tables and results

Supplementary Table 1 shows all bit configurations for the 8 OTTDLs, from ΔT = -16 U to ΔT = 15 U. The first row corresponds to the value of the delay in each line and the second row presents the corresponding precompensation.

	Line 1	Line 2	Line 3	Line 4	Line 5	Line 6	Line 7	Line 8
**Line delay (τ)**	1U	2U	3U	4U	5U	6U	7U	8U
**Precompensation**	112U	96U	80U	64U	48U	32U	16U	0U
**Bit (00000)**	112U	96U	80U	64U	48U	32U	16U	0U
**Bit (00001)**	113U	98U	83U	68U	53U	38U	23U	8U
**Bit (00010)**	114U	100U	86U	72U	58U	44U	30U	16U
**Bit (00011)**	115U	102U	89U	76U	63U	50U	37U	24U
**Bit (00100)**	116U	104U	92U	80U	68U	56U	44U	32U
**Bit (00101)**	117U	106U	95U	84U	73U	62U	51U	40U
**Bit (00110)**	118U	108U	98U	88U	78U	68U	58U	48U
**Bit (00111)**	119U	110U	101U	92U	83U	74U	65U	56U
**Bit (01000)**	120U	112U	104U	96U	88U	80U	72U	64U
**Bit (01001)**	121U	114U	107U	100U	93U	86U	79U	72U
**Bit (01010)**	122U	116U	110U	104U	98U	92U	86U	80U
**Bit (01011)**	123U	118U	113U	108U	103U	98U	93U	88U
**Bit (01100)**	124U	120U	116U	112U	108U	104U	100U	96U
**Bit (01101)**	125U	122U	119U	116U	113U	110U	107U	104U
**Bit (01110)**	126U	124U	122U	120U	118U	116U	114U	112U
**Bit (01111)**	127U	126U	125U	124U	123U	122U	121U	120U
**Bit (10000)**	128U	128U	128U	128U	128U	128U	128U	128U
**Bit (10001)**	129U	130U	131U	132U	133U	134U	135U	136U
**Bit (10010)**	130U	132U	134U	136U	138U	140U	142U	144U
**Bit (10011)**	131U	134U	137U	140U	143U	146U	149U	152U
**Bit (10100)**	132U	136U	140U	144U	148U	152U	156U	160U
**Bit (10101)**	133U	138U	143U	148U	153U	158U	163U	168U
**Bit (10110)**	134U	140U	146U	152U	158U	164U	170U	176U
**Bit (10111)**	135U	142U	149U	156U	163U	170U	177U	184U
**Bit (11000)**	136U	144U	152U	160U	168U	176U	184U	192U
**Bit (11001)**	137U	146U	155U	164U	173U	182U	191U	200U
**Bit (11010)**	138U	148U	158U	168U	178U	188U	198U	208U
**Bit (11011)**	139U	150U	161U	172U	183U	194U	205U	216U
**Bit (11100)**	140U	152U	164U	176U	188U	200U	212U	224U
**Bit (11101)**	141U	154U	167U	180U	193U	206U	219U	232U
**Bit (11110)**	142U	156U	170U	184U	198U	212U	226U	240U
**Bit (11111)**	143U	158U	173U	188U	203U	218U	233U	248U

Supplementary Table 1. All the configurations of the beamforming network together with the delay value in each line and the corresponding precompensation value.

Supplementary Note 3.

Phase delay and group measurements per OTTDL unit

The phase and time delay measurements were obtained as indicated in the Methods Section. Below, in Supplementary Figure 4, there are the phase and group delay results for the 32 different bit combinations in each one of the eight lines.

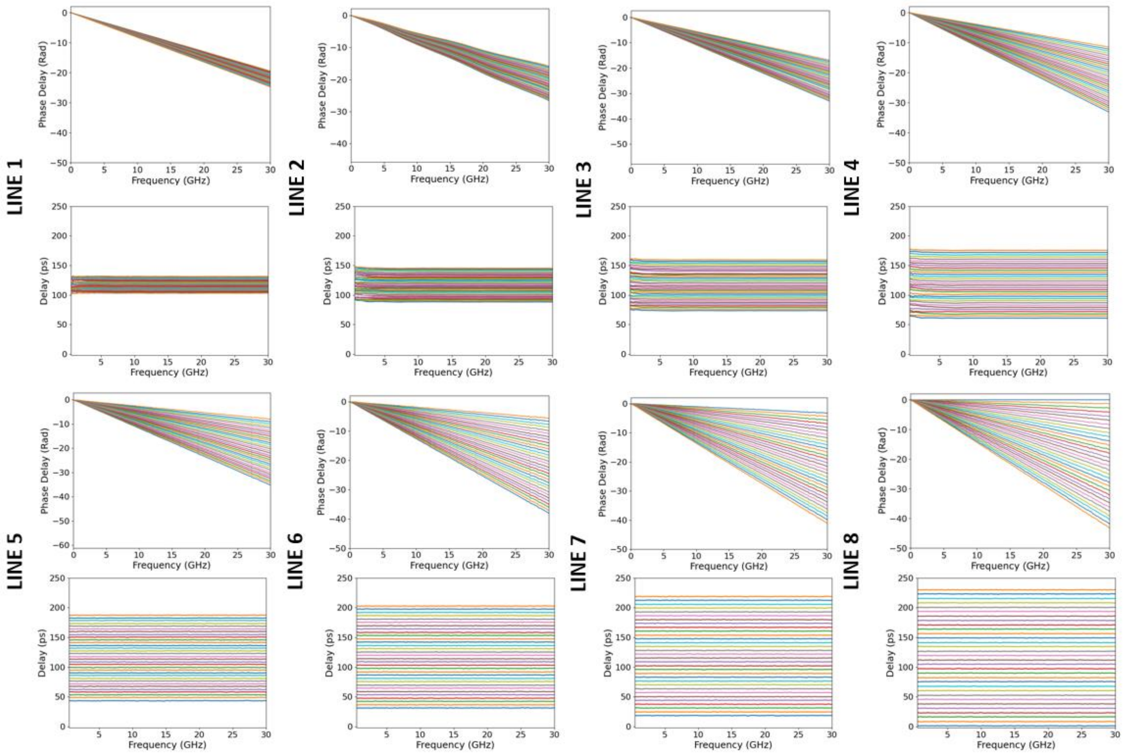

Supplementary Figure 4. Phase and group delay all the lines and bit configurations.

In the first line, the time delays for all bit configurations are relatively close to each other, primarily because the delay per stage in this line is the smallest of the entire array. This, in turn, affects the phase delay, resulting in similar slopes for all the configurations. These measurements change with every line up to the eighth, where the delay is maximum, and we can see a big difference in time and slopes for every bit word. All measurements were taken using as reference the smallest optical path within the beamforming network, the eighth line for bitword 00000.

The value of the precompensation delay in each line can be appreciated, from which the time delay starts to grow as the bit words increase. The delay value for each line in all the configurations of the beamforming network is represented in the following graphs, and it can be seen in a very visual way how the precompensation delay allows to obtain increasing delays in the two directions of the array.

Supplementary Fig. 5 shows the delays in each line for the first set of 4 bit word configurations (0000 to 0111) together with the corresponding beam pattern for each configuration. Supplementary Fig. 6 shows identical information for the second set of 4 bit word configurations (1000 to 1111).

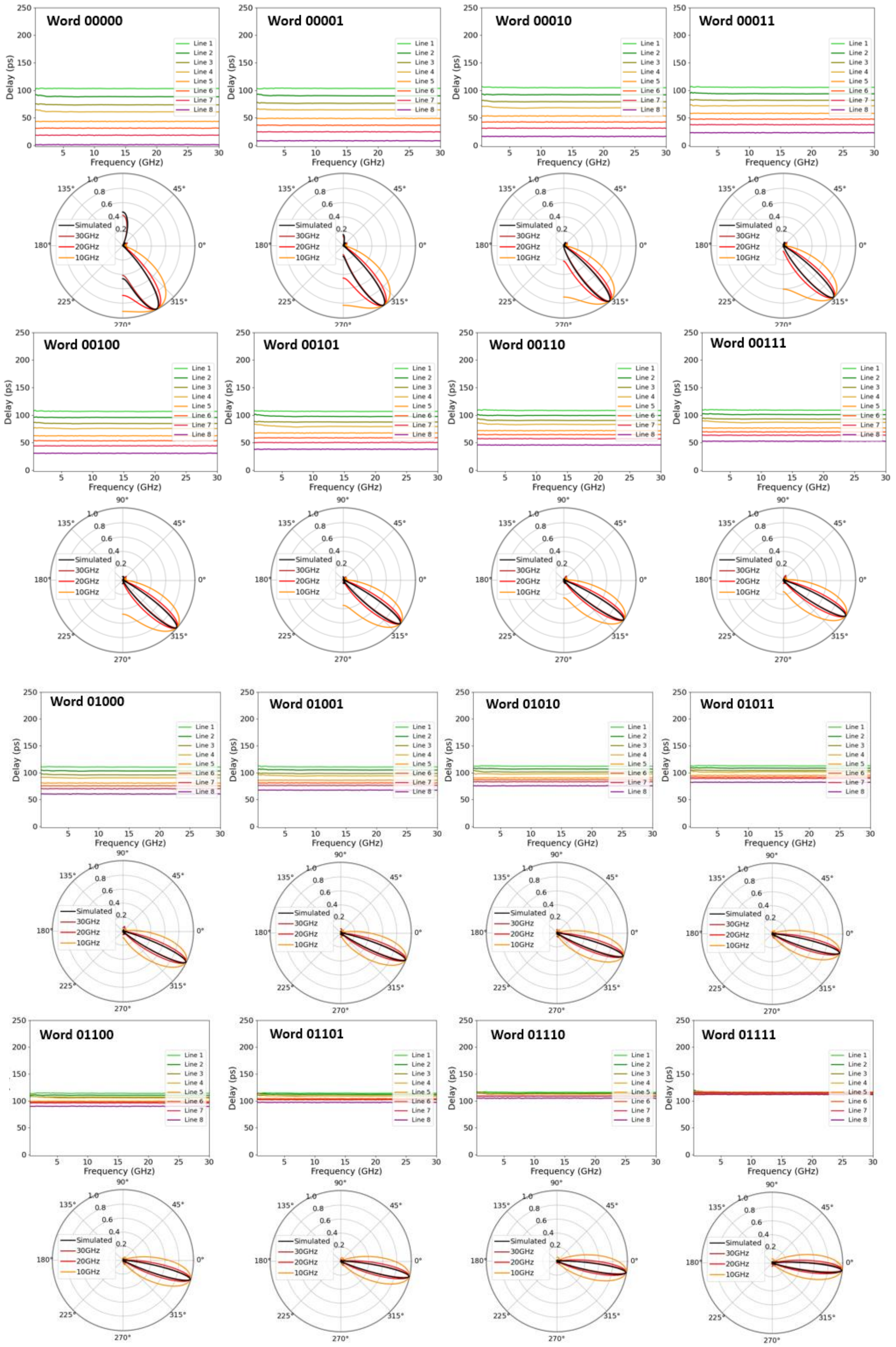

Supplementary Figure 5. Delays and beam patterns for the first set of 16 different pointing angles (bit words 0000 to 0111).

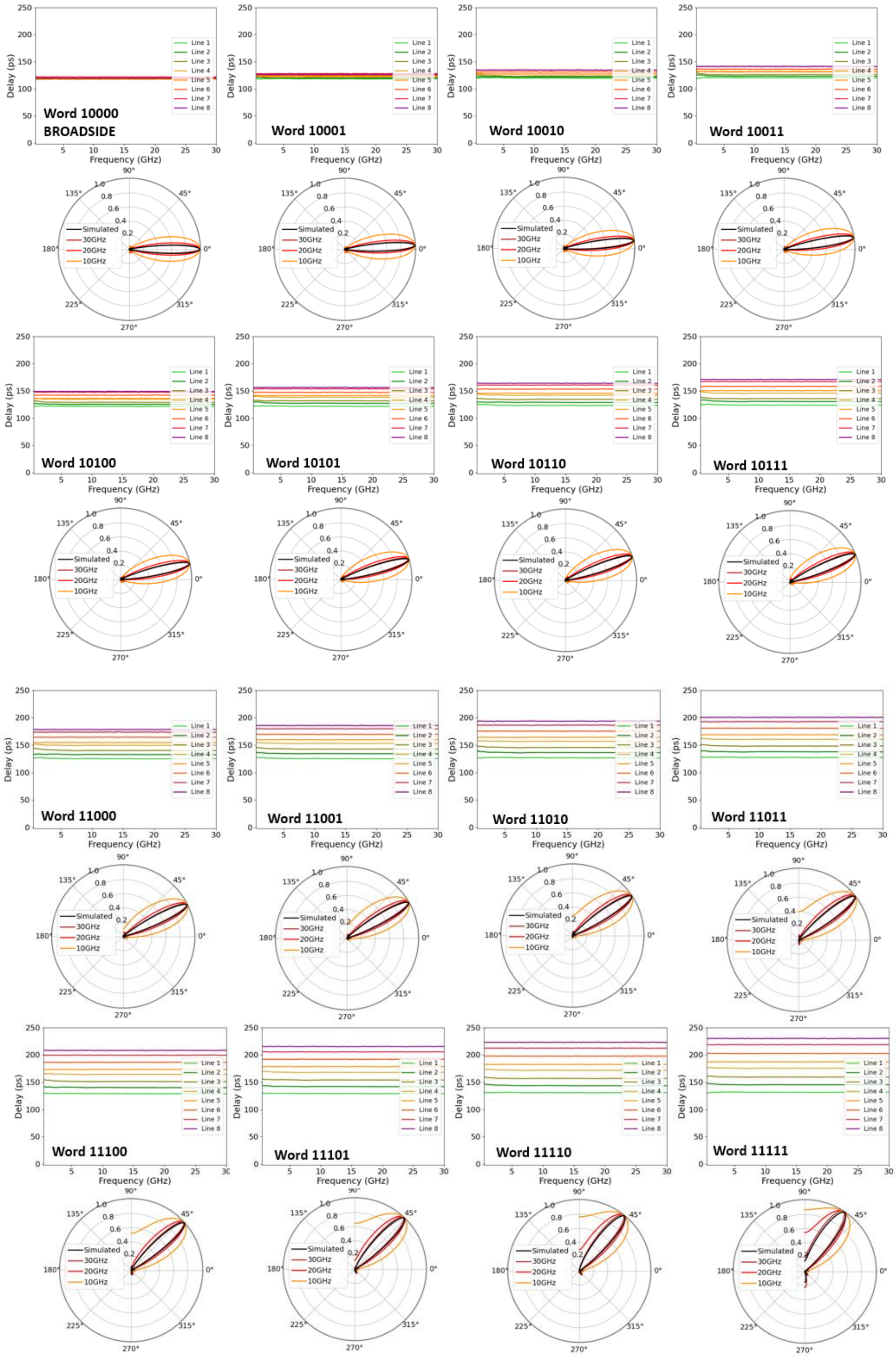

Supplementary Figure 6. Delays and beam patterns for the second set of 16 different pointing angles (bit words 1000 to 1111).

The system starts pointing to negative angles, with a decreasing delay from the first line to the eighth one. As the bit word increases, the relative delay between lines becomes smaller until reaching the bit word 10000, where all lines suffer the same delay, and we obtain the broadband configuration.

After this, the temporal separation between lines grows again, but this time in the opposite direction of the array, allowing pointing at positive and negative angles.

Supplementary Note 4

Power Consumption

The power consumption for the beamforming network comes from the phase actuators based on thermo-optics form the switchable delay lines. Table 1 shows the power consumption for the bar and cross states of all the tunable couplers on the chip (including the optional pre-compensation stage). The consumptions for the cross and bar states are different for each switch due to differences in the manufacturing process but in general the power consumption for a π-phase shift is around 1.29 mW. The average consumption per line is approximately 9 mW giving a total consumption for the complete optical beamforming network of 72 mW.

Supplementary Table 2 compiles all the consumption values for the two positions of the optical switches, 7 for each delay line.

Switch	Cross (mW)	Bar (mW)	Switch	Cross (mW)	Bar (mW)
S_{10}	0	1.51	S_{20}	0	2.42
S_{11}	2.45	1.14	S_{21}	1.06	2.38
S_{12}	0.07	1.39	S_{22}	0.90	2.27
S_{13}	0.31	1.60	S_{23}	0.91	2.23
S_{14}	0.65	1.95	S_{24}	0.94	2.27
S_{15}	2.45	1.15	S_{25}	0.80	2.05
S_{16}	1.17	2.45	S_{26}	0.32	1.59
S_{30}	0	1.35	S_{40}	0	1.78
S_{31}	2.50	1.20	S_{41}	0.70	20.3
S_{32}	2.27	0.99	S_{42}	0.60	1.93
S_{33}	2.16	0.87	S_{43}	0.57	1.84
S_{34}	0	1.28	S_{44}	0.23	1.50
S_{35}	0.15	1.49	S_{45}	0.03	1.34
S_{36}	1.50	0	S_{46}	1.52	0
S_{50}	0	1.98	S_{60}	0	0
S_{51}	1.78	0.51	S_{61}	0.18	1.49
S_{52}	2.08	0.78	S_{62}	2.10	0.77
S_{53}	2.11	0.78	S_{63}	2.21	0.93
S_{54}	1.44	0.16	S_{64}	1.96	0.68
S_{55}	1.67	0.36	S_{65}	2.09	0.79
S_{56}	2.25	2.25	S_{66}	2.16	0.82
S_{70}	0	1.01	S_{80}	0	1.51
S_{71}	2.07	0.78	S_{81}	1.35	0
S_{72}	0.69	2.03	S_{82}	0.80	2.09
S_{73}	0.45	1.73	S_{83}	0.52	1.88
S_{74}	0.60	1.92	S_{84}	1.27	0
S_{75}	0.65	1.88	S_{85}	0.63	1.98
S_{76}	2.07	0.76	S_{86}	1.14	0.01

Supplementary Table 2. Compilation of the power consumption of the MZIs corresponding to each OTTDL stage.

Once the correct operation of the architecture has been checked, power consumption can be reduced if the precompensation stage is fixed instead of having a specific delay stage for it.

Supplementary Note 5

Effect of loss imbalance in the Array Factor

Since the delay line stages in the beamformer have different waveguide lengths one may ask whether these can yield significant differences in propagation losses, which might potentially induce an undesired apodization effect in the beam pattern. Here we report the main simulation results of our architecture for different waveguide loss coefficients, ranging from 0 to 5 dB/cm (including our measured value of 1.1 dB/cm). Losses>3 dB/cm are far beyond the worst-case scenario on any foundry nowadays, but we have nevertheless included them to prove that the apodization effect is negligible.

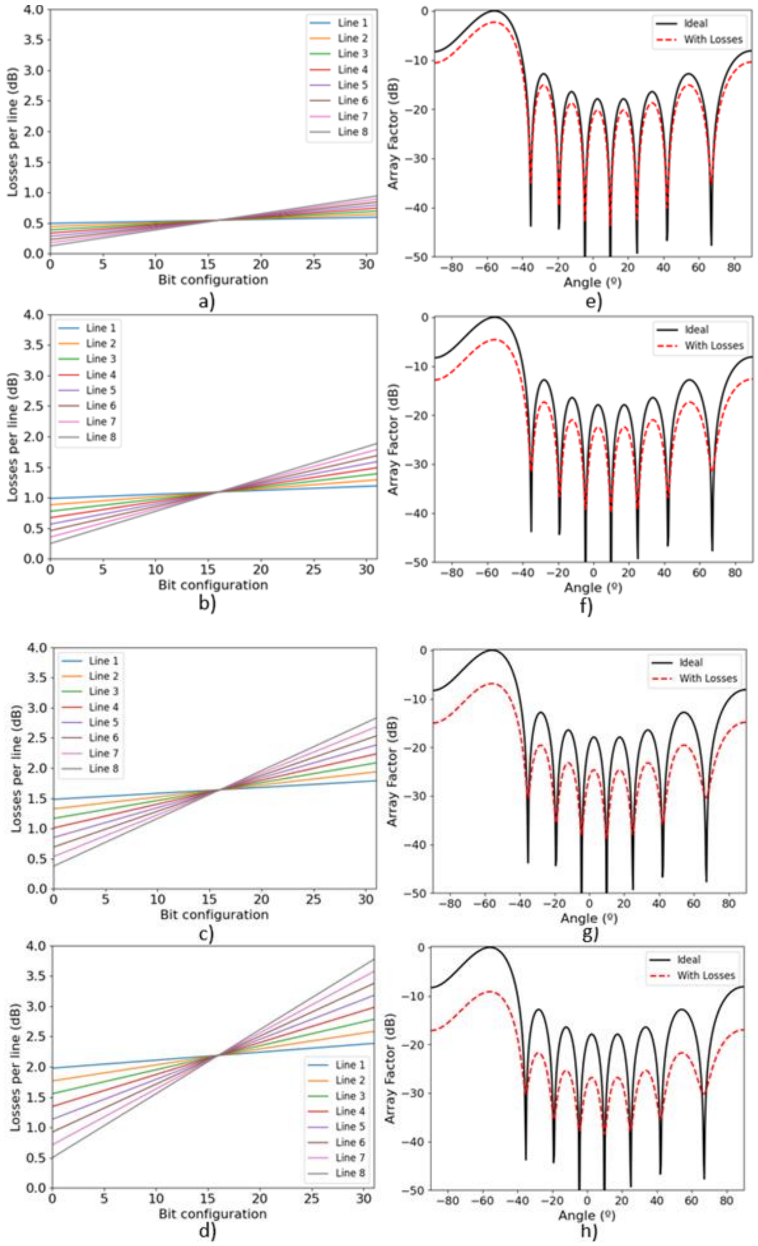

Supplementary Figure 7. **(a-d)** Losses per line for 0.75, 1.5, 2.25 and 3 dB/cm as loss coefficient. **(e-h)** The corresponding side angle (bitword 00000).

Supp. Figure 7 (a-d) illustrates the effect of various waveguide propagation loss values in each of the delay lines across all the 32 5-bit word configurations. Due to the precompensation stage,

the most substantial dissimilarities for the extreme side angles, whereas for the broadside configuration, all lines exhibit uniform propagation losses. Therefore, in Supp. Fig. 6 (e-h) we plot the corresponding beam patterns for one of the two extreme side angles (-60º) Inevitably, as the propagation losses increase the directivity and the sidelobes' extinction ratio decrease. However, in terms of its shape, the pattern closely retains its ideal form compared to that obtained with a uniform apodization, even in the presence of losses. In Supp. Fig. 8 we show the variation of the Main Lobe to Secondary Ratio (MLSR) with the loss coefficient for the worst-case extreme side pointing angle, proving that the value of tapering is negligible.

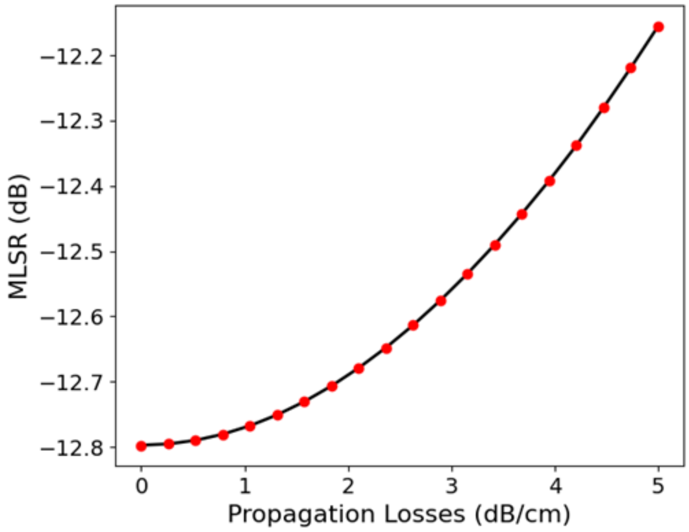

Supplementary Figure 8. The value of the MLSR for different propagation losses. In order to observe an appreciable change, we have to allow for propagation losses much higher than those routinely attainable in the current state of the art.

Supplementary Note 6

Before measuring the beamforming network, we collected data to estimate the performance of the fundamental building blocks employed in the final design, in order to characterize their insertion losses and wavelength dependence.

All the structures show a flat response in wavelength and the fittings were done for the principal wavelength of use, 1550 nm.

Propagation Losses measurements

We used the cutback technique to measure the propagation losses in straight waveguides of different lengths incorporated as test structures in the chip as shown in Supplementary Fig. 9

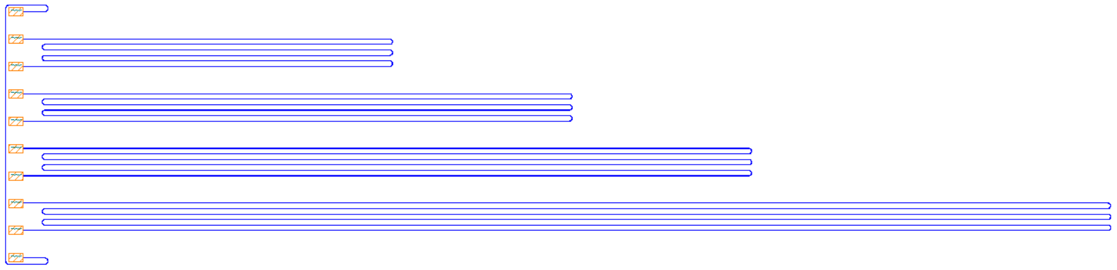

Supplementary Figure 9. Structure used to measure the propagation losses of the waveguides. Input by grating coupler array.

The main results for the spectral dependence of losses in the 1520-1580 nm wavelength range are shown in the left part of Supplementary Fig. 10, where each color corresponds to a waveguide of different lengths. From those, we can extrapolate for a given wavelength a linear regression curve as shown in the right part of Supplementary Fig. 10 for 1550 nm. At this wavelength the slope corresponds to a loss of 1.1 ± 0.3 dB/cm.

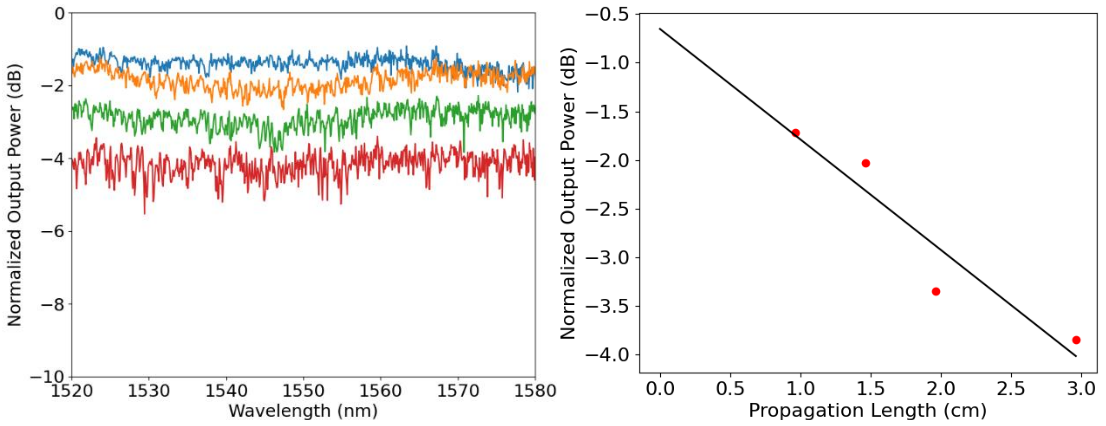

Supplementary Figure 10. Wavelength spectrum and linear fitting for the different lengths. The responses correspond to each of the different lines, each one longer than the previous one. The propagation loss coefficient obtained is 1.1 ± 0.3 dB/cm.

MMI Losses measurements

Similarly, we used a set of test MMI configurations as shown in Supplementary Fig 11 to allow us to measure the insertion losses of a cascade of MMIs ranging from 1 to 10.

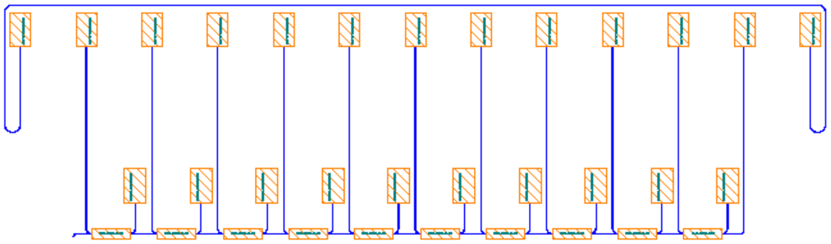

Supplementary Figure 11. Structure used to measure the insertion losses of the MMIs. Input by grating coupler array.

The main results for the spectral dependence of losses in the 1520-1580 nm wavelength range are shown in the left part of Supplementary Fig. 12, where each color corresponds to a cascade of different numbers of MMI couplers. From there, we can extrapolate for a given wavelength a linear regression curve as shown in the right part of Supplementary Fig. 10 for 1550 nm. At this particular wavelength, the slope corresponds to an insertion loss of 0.15 ± 0.05 dB/MMI.

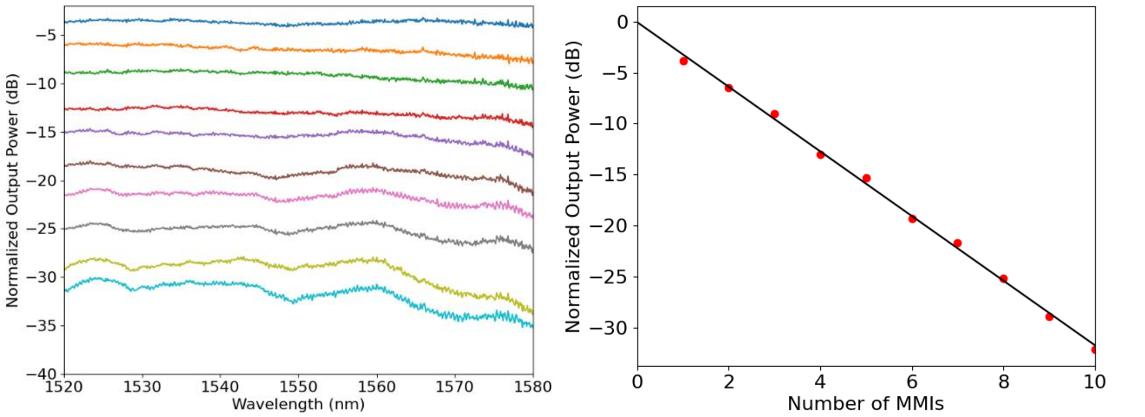

Supplementary Figure 12. Wavelength spectrum and linear fitting for a different number of concatenated MMIs, from 1 to 10. The slope analysis provided us with the insertion loss value, obtained after accounting for the power loss resulting from the MMI's 50/50 splitting ratio. Our measurement indicates an insertion loss of 0.15 ± 0.05 dB/MMI.

MZI measurements

Finally, we characterized the insertion losses for different switching MZI units employed to setup the signal routing in each delay line according to the programmed bit word. The main insertion losses versus the wavelength results are shown in Supplementary Fig. 13, where the different curves correspond to different currents applied to the thermo-optic actuators (0 to 0.7 mA). The extreme values correspond to bar and cross states while the intermediate correspond to partial coupling. As in the previous cases, the results at 1550 nm were employed to find the MZI insertion loss at that wavelength yielding a value of 0.45 dB.

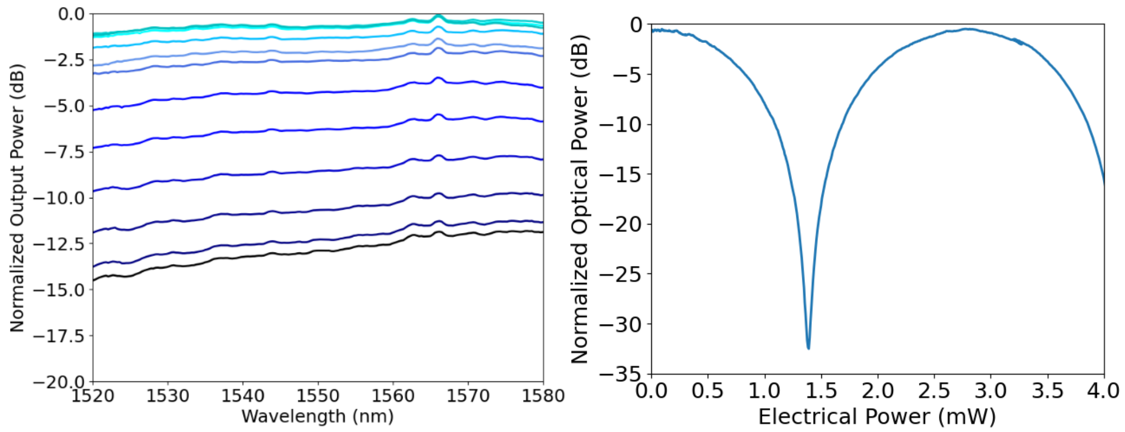

Supplementary Figure 13. The colored lines show the wavelength response for different currents applied to the thermo-optic actuators. On the right side, the response of one of the MZI at 1550 nm.
